# Speech correlates of neuropsychiatric symptoms in older adults

**DOI:** 10.1002/alz.095361

**Published:** 2025-01-09

**Authors:** Laili Soleimani, Nadia Ramsingh, Sunghye Cho, Yoon Duk Kim, Carmen Gonzalez‐Recober, Arash Kia, Carolyn W. Zhu, Jimmy Akrivos, Judith A. Neugroschl, Hillel Grossman, Faye Sheppard, Truda Silberstein, Mark Y Liberman, Michal S Beeri, Mary Sano, Naomi Nevler

**Affiliations:** ^1^ Icahn School of Medicine at Mount Sinai, New York, NY USA; ^2^ Linguistic Data Consortium, University of Pennsylvania, Philadelphia, PA USA; ^3^ Penn FTD Center, University of Pennsylvania, Philadelphia, PA USA; ^4^ James J. Peters VA Medical Center, Bronx, NY USA; ^5^ Icahn School of Medicine at Mount Sinai, NEW YORK, NY USA; ^6^ Herbert and Jackeline Krieger Klein Alzheimer’s Research Center, Rutgers Biomedical and Health Sciences, Newark, NJ USA

## Abstract

**Background:**

Neuropsychiatric symptoms (NPS) including depression and apathy, are common in dementia and profoundly affect both patients and caregivers. These symptoms can manifest early and can be indicative of the disease progression. The existing tools for measuring NPS lack objectivity and are not sensitive enough to detect subtle changes in the earlier stages of dementia. Here, we report a preliminary analysis of the associations of speech markers with NPS in older adults from the Mount Sinai Alzheimer’s Disease Research Center (ADRC).

**Method:**

Participants aged 60 and above were recruited through the Mount Sinai ADRC. Cognition was measured by the Montreal Cognitive Assessment (MOCA), and NPS by the Geriatric Depression Scale (GDS) and the Neuropsychiatric Inventory (NPI‐Q). Speech samples, collected during description of the “cookie theft” picture. Samples were then transcribed manually and processed using automated acoustic and lexical pipelines.

Nonparametric partial correlation analysis was used to examine the relationship of the speech markers with the behavioral measures, adjusting for age, sex, education and MOCA score.

**Result:**

Participants (N = 54) averaged 79 ± 7.73 years of age, 55.6% were female. They had a mean MOCA score of 24.91 (±4.08), GDS score of 2.6 (±2.59) and NPI‐Q score of 2.17(±3.21).

Higher GDS scores, indicative of more severe depression, were associated with higher mean pitch values (r = +0.376, p = 0.015). Higher NPI‐Q scores, reflecting greater NPS severity, were associated with reduced speech complexity, including using more incomplete (r = + 0.350, P = 0.025) and less‐novel (r = +0.313, p = 0.046) words. Higher NPI‐Q scores were also correlated with words with higher valence (i.e., more pleasant content, r = +0.363, p = 0.02) and a tendency toward more dominant speech (r = +0.297, p = 0.059).

**Conclusion:**

Our preliminary results so far show the potential of speech (both acoustic and lexical features) as a marker for NPS. Specifically, we observed relationships between speech characteristics and self‐reported depression symptoms, as well as NPI scores reflecting symptom severity. Further research is necessary to validate and refine these associations, ultimately paving the way of speech analysis as a promising non‐invasive objective method for detecting subtle behavioral changes in the early stages of dementia.

